# Case of Intestinal Obstruction, Caused by Stricture of the Ascending Colon

**Published:** 1830-02

**Authors:** R. S. Hutchinson

**Affiliations:** Member of the Royal College of Surgeons, London, and of the Royal Medical Society of Edinburgh, &c.


### INTESTINAL OBSTRUCTION.

*Case of Intestinal Obstruction, caused by Stricture of the ascending Colon.* By R. S. Hutchinson, m.d. Member of the Royal College of Surgeons, London, and of the Royal Medical Society of Edinburgh, &c.

Master H., ætat. eleven, a pupil in the academy of the Rev. C. Fletcher, on the morning of the 16th September, first complained to one of his schoolfellows of indisposition, having a slight headach; but he ate on that day a very hearty dinner, and played in the afternoon for some hours at cricket with great alacrity, and in the evening appeared perfectly well. On the morning of the 17th, he again complained that his head ached, and after breakfast was sick, rejecting only what he had taken. A gentle aperient was given him, which, however, did not operate; and Mr. Wanich, member of the Royal College of Surgeons, was sent for to see him, who ordered a pill with four grains of calomel to be taken at night, and a common aperient draught early in the morning. The draught did not remain upon the stomach; but, after taking it, a small and somewhat costive motion was passed, and the boy expressed himself as much better; but his bowels not being again acted upon, he was ordered a repetition of the calomel.

At six o'clock on the morning of the 19th, he first complained of some pain in the bowels; when he was bled to about seven ounces; castor oil and other aperients were administered, which produced, in the middle of the day, a small, watery evacuation.

The pain continuing until the evening, I was requested to see him, and at six o'clock found him with the following symptoms: He complained of pain, not generally over the abdomen, but principally situated on the left side, not increased on pressure; the belly felt somewhat hard and distended. On examining the external abdominal ring and umbilicus, I found no appearance of hernia. The pulse was beating 110 in a minute, small, and easily compressed, resembling that of a person who had recently suffered some sudden injury or shock, before reaction takes place. The tongue was generally whitish, no redness either at the tip or edges; the countenance pallid, and rather anxious; skin cool. The pain was not acute, but productive of great restlessness; the sensorium perfectly undisturbed. Blood drawn in the morning exhibited no appearance of inflammation. He was ordered, after the warm bath, hot fomentations to the belly; bottles of hot water to the feet; a few spoonsful of strong beef-tea occasionally; and to take, every half-hour, two grains of calomel, with four of compound extract of colocynth.

Eleven o'clock P.M.—Remained in the same state: bowels not acted upon; medicine and beef-tea retained. A solution of one ounce and a half of Epsom salts, in six ounces of Inf. Sennæ, was prescribed, two spoonsful of which were to be taken with each dose of calomel and colocynth; an injection, consisting of a pint of gruel and half an ounce of castor oil, to be administered every two hours.

These medicines were continued during the night, the stomach retaining them. A greater part of the injections passed away, but unmixed with any feculent matter; nor did the medicines at all act upon the bowels.

On the morning of the 20th, the abdomen was hard, tumid, and generally painful; no relief experienced from the fomentations; now tender on pressure, and disliking the flannel, which was used as a vehicle for the hot application. I therefore changed it, and applied over the belly a bag containing scalded bran; the weight of which, however, the patient could not bear. Pulse 124, smaller, and more thready; warmth more perfectly re-established over the whole body; restlessness increased; the countenance more anxious; eye intelligent, and sensorium free.

Eight o'clock A.M.—I gave him, with ten grains of calomel, two drops of croton oil, which were repeated every half-hour, until he had taken eight drops. About noon, slight sickness came on, which was generally suppressed by the timely application of pressure upon the stomach. He was troubled also with slight hiccup. Since ten o'clock, small quantities of wine and water had been administered, and occasionally doses of æther, when the hiccup was most troublesome.

Twelve o'clock.—I ordered a mixture with an ounce and a half of Sp. Terebinth. mixed with six ounces of peppermint water, by means of the yolk of an egg; three tablespoonsful to be taken every honr; and an injection with one ounce of the turpentine and a quart of gruel, to be immediately administered.

At this time another medical man was called in consultation, who supposed that the obstruction of the bowels might be caused by an insidious inflammation, and that the pulse would be found to rise, and become more full, by the abstraction of a small quantity of blood. Ten leeches were ordered to be applied to the belly, and be allowed to remain, if the pulse should appear to warrant their continuance. After being applied a few minutes, the pulse became evidently affected and sinking, and the patient faint: they were immediately removed, and the bleeding suppressed by continued pressure kept upon the orifices. Some brandy and water was given, but the pulse continued low and feeble, and our little patient appeared evidently and gradually sinking. Clysters with turpentine and large quantities of fluids were injected, with much force, by the patent syringe, to endeavour mechanically to overcome the obstruction: the bowels, however, remained unacted upon. The patient continued perfectly collected, but the vital powers gradually sinking. Sickness did not come on until about seven o'clock in the evening: when in the act of raising himself suddenly to vomit, and saying he felt as if he could scarcely speak, he sunk down on his back, and died, thirty-seven hours from the commencement of the pain in the belly.

*Examination of the body, forty hours after death.*—The corpse externally presented the appearance of a remarkably fine youth, who had died suddenly. No great tumefaction or hardness of the belly.

On opening the abdomen, no marks of general inflammation appeared; but from two to three inches of the lower portion of the ilium, on the peritoneal coat, the vessels were much injected, and some shreds of coagulating lymph were attached to it; the mucous membrane in the same situation was slightly reddened. There were no adhesions between any portions of the small intestines. Upon tracing the intestinal canal along the colon, the cause of all the mischief became evident. At the upper part of the ascending colon, about two inches before that intestine commenced to cross the abdomen, and form its transverse arch, a band, or prolongation of the peritoneum was seen to cross from one side of the gut to the other, attached on the right side to that part of the peritoneum which, passing from the ascending colon, is continued lining the abdominal muscles, to the opposite or left side of the intestine. Thus this portion of the ascending colon was included in a ligature, almost as perfect as if a string had been tightly bound around it. The band which formed the stricture was about six lines in width, uncommonly strong, and not injected with red blood-vessels, but having exactly the same appearance as a healthy portion of peritoneum. The intestine in its immediate neighbourhood was only very slightly inflamed, and no evidence of inflammatory action existed in any other portion of the large intestine. Before the stricture was divided, the opening left by it in the colon was so small as not to admit the tip of the little finger, or even a good-sized quill; but when the band was cut through, the intestine, by the elasticity of its coats, resumed its natural caliber. No thickening of the coats had taken place, nor was there any deposit between them. The cæcum was distended by the presence of the ingesta, and its contents smelt strongly of turpentine. No scybala were found in any part of the canal. The rest of the abdominal viscera were perfectly healthy.

It will be found very difficult to give a satisfactory explanation how the stricture in this case was formed. If it was the product of inflammatory action, a short period only can have been allowed for its appearance. On the 16th of September, the patient was well enough to play actively at cricket; on the 18th and 19th, the bowels were acted upon, though but slightly; on the 20th, he died. The band itself had no appearance of lymph which has become organised, but in every respect resembled in appearance healthy peritoneum, from whence it seemed to be a mere elongation or process. Nor can this case, I fear, be adduced as any guide to us in future practice; for although, if we could have positively ascertained the precise situation of the stricture, its division might have been attempted with as much safety as in a case of strangulated hernia, and would have, I am convinced, ensured success if early performed, yet no guide to its situation existed: the pain was principally referred to the left side, although the seat of stricture was in the ascending colon, and of course on the right. I have been induced to offer this case to the consideration of my medical brethren, as one which I have not found recorded in medical works, and as a pathological fact which probably may explain some otherwise obscure case of intestinal obstruction, when positive means of ascertaining the truth may not be allowed.

That this is not a solitary instance of intestinal obstruction from a similar cause, I have had an opportunity of knowing; for some years back, when I was demonstrator in the Webb-street School of Anatomy, I was requested by Dr. Southwood Smith to go with him to examine the body of a woman, housekeeper to one of the city aldermen, who, he told me, had long since laboured under supposed chronic disease of the liver, but for three days had suffered from obstruction of the bowels, which had resisted every means employed to overcome it.

The subject had been a woman of about sixty years of age. The abdomen was greatly distended, and very hard, with much fulness on the right side, as from a greatly enlarged liver. Upon opening the abdomen, the right side of the transverse arch of the colon started into view, distended to an enormous extent, and capable of containing many quarts. From the under part of the greater curvature of the stomach, and near to its cardiac extremity, a band, similar in most respects to the one I have attempted to describe in the former case, passed downwards, and surrounded the portion of the transverse arch of the colon immediately beneath it, forming a firm, unyielding stricture: beyond this, the intestine was healthy and empty; the liver undiseased, and not enlarged. In this case no thickening of the coats, nor deposition between them, existed.

*Southwell, Notts.; December 28th,* 1829.

